# Miscellaneous Medical Intelligence

**Published:** 1850-09

**Authors:** 


### ARTICLE VI.

### MISCELLANEOUS MEDICAL INTELLIGENCE.

*Medical Appointments.*—S. Hanbury Smith, late Professor of Practice, in Starling medical college, and Editor of the *Ohio Med. and Surg. Journal*, has been appointed Superintendent of the Ohio Lunatic Asylum, in place of the distinguished Dr. Awl, resigned.

Prof. S. M. Smith has been transferred from the chair of Materia Med. to that of Practice, vacated by the resignation of Prof. S. Hanbury Smith, in the Starling medical college, and Prof. Charles A. Lee, of New York, has succeeded to the chair thus vacated.

Dr. John Bell, of Philadelphia, one of the most distinguished authors of our country, has been appointed to the chair of Practice in the medical college of Ohio, resigned by Dr. Drake.

All the chairs in the medical college of Ohio were vacated by the Board of Trustees, after the resignation of Dr. Drake, and Drs. Mussey, Locke, Lawson and Shotwell were re-appointed, and Dr. Landon C. Rives was appointed to the chair of Obstetrics, &c., and Dr. T. O. Edwards to that of *Materia Medica*.

Dr. Meeker has been appointed to the chair of Anatomy, and Dr. Deming to that of Special Pathology and Institutes of medicine, in Ind. Central medical college, at Indianapolis.

Prof. Geo. B. Wood has been transferred from the chair of materia medica to that of Practice, resigned by Prof. Chapman, in the University of Pennsylvania, and Dr. Joseph Carson is appointed to the chair thus vacated. These are excellent appointments.

The distinguished Dr. Mott resigned the chair of Surgery in the University of New York, on account of the election of Dr. Detmould to the chair of Practice, lately resigned by Dr. Dickson, whereupon Dr. Detmould also resigned. Profs. Gross and Bartlett, of the University of Louisville, Ky., have been appointed to these chairs, of course leaving two vacancies in the latter school. Dr. Dickson has been re-appointed to a chair in the medical college of South Carolina, at Charleston.

The department of Physiology has been transferred to the Chair of Anatomy in Rush medical college, and that of Clinical medicine instituted, so that the chair occupied by Prof. Davis is Pathology and clinical medicine.

Dr. E. N. Horsford, has been appointed to the chair of Chemistry in Harvard University, lately occupied by Prof. Webster.

Dr. James Bryan has been appointed to the chair of Institutes of medicine and medical Jurisprudence in the Philadelphia college of medicine.

*Kinesipathy.*—A new pathy has been invented by a Swede named Ling, under the not very euphonious name of Kinesipathy. It proposes to cure diseases by some kind of gymnastic exercises. It promises to rival Homeopathy, Hydropathy, Isopathy, &c.

The latest mode of raising the wind by a physician is said to be, by sending a crier through the streets with a drum to call attention, offering a large reward for a lost dog; on his return to Dr. ——, at his lodgings. The gossips will take the idea that the Doctor is rich and a great physician, and so his name and place will be advertised.

Dr. Bond, of Philadelphia, has invented an improvement of the Obstetrical Forceps, by which the branches are more readily locked, and while the clams may not be precisely opposite to and facing each other. It is a good idea.

M. Delioux lately read a paper before the French Academy of *Medicine* on the use of Chloroform as an anti-periodic in obstinate intermittent Fevers. He gives 30 or 40 drops in throe doses, near together, so that the last shall be taken about three hours before the paroxysm is expected.

It is suggested by the *N. Y. Medical Gazette* that when doctors fight duels, the weapons used should be pills. We fear the suggestion will alarm its non-professional readers.

A new substitute for Quinine, called Quinoa, which is a plant cultivated in Peru, is proposed by *D*r. Buckler, of Baltimore.

Sixty-nine gentlemen were graduated on the 19th of July last, by the Philadelphia college of medicine.

It is reported that Leibig, the distinguished chemist is about to visit the United States.

Kousso, a new remedy for tape-worm, is said by the *London Lancet* to be very efficient, so far as tried.

A tart controversy is going on between the *Western Journal of Medicine and Surgery*, and the *Transylvania Medical Journal*, in reference to the new medical school at Louisville. The former tries to show that this school has no legal existence; the latter that it has.

A fatal case of poisoning recently occurred in Boston, in consequence of an apothecary filling a prescription for 10 grains Calomel with Corrosive Sublimate. The apothecary has been arrested, and awaits a trial for manslaughter under bail of $5,000.

Paine's discovery that gas can be made cheap from water, is generally regarded as a hoax: as well as his new theory that water is a simple, and that oxygen and hydrogen gases are water combined with positive and negative electricity.

